# Identification and Characterization of the Roles of circCASP9 in Gastric Cancer Based on a circRNA-miRNA-mRNA Regulatory Network

**DOI:** 10.1155/2022/9416825

**Published:** 2022-03-14

**Authors:** Chuan Qin, Han Zhang, Xiong Guo, Anqi Cheng, Huawen Liu, Ziwei Wang

**Affiliations:** ^1^Department of Gastrointestinal Surgery, The First Affiliated Hospital of Chongqing Medical University, Chongqing, China; ^2^Department of Gastrointestinal Surgery, Chongqing University Three Gorges Hospital, Chongqing University, Chongqing, China; ^3^Department of Oncology, Chongqing University Three Gorges Hospital, Chongqing University, Chongqing, China

## Abstract

Accumulating evidence demonstrates that circular RNAs (circRNAs) have substantial effects on gastric cancer (GC) tumorigenesis and development. In this study, we performed a screen and identified two differentially expressed circRNAs (circCASP9 and circDLG5) from our circRNA microarray. We validated the expression of circCASP9 and circDLG5 in GC tissues and their normal counterparts by using qRT-PCR. Only circCASP9 was revealed to be downregulated in tumor tissues compared with adjacent normal tissues. Functionally, circCASP9 significantly inhibited the proliferation, migration, and invasion of GC cells both *in vitro* and *in vivo*. A competing endogenous RNA (ceRNA) network was constructed for the identification of candidate target genes of circCASP9. circCASP9, two miRNAs, and 55 mRNAs were selected for construction of the ceRNA network. We confirmed that circCASP9 can function as a sponge of miR-589-5p to regulate KANK1 expression, thereby controlling GC progression. Accordingly, we identified that the novel circRNA circCASP9 was differentially expressed between GC tissues and their normal counterparts. We also showed that circCASP9 can regulate the growth and metastasis of GC via the miR-589-5p/KANK1 axis. The circCASP9/miR-589-5p/KANK1 axis might provide crucial insights for investigating the occurrence and development of GC.

## 1. Introduction

Gastric cancer (GC) is an important cancer worldwide, with more than 1 million new cases in 2020 and with the fifth highest incidence (5.6%) after breast cancer (11.7%), lung cancer (11.4%), colorectal cancer (10.0%), and prostate cancer (7.3%). According to the latest data regarding the global burden of cancer, GC is estimated to cause 769,000 deaths annually, with the fourth highest mortality rate (7.7%) after lung cancer (18%), colorectal cancer (9.4%), and liver cancer (8.3%) [1]. Since the onset of GC is more insidious than that of other cancers, the symptoms are not typical, and there is still a lack of effective early biomarkers, it is often in the middle or late stage at diagnosis, which makes its prevention and treatment highly challenging [2, 3]. Although the current comprehensive treatment regimen mainly includes surgery, chemotherapy, and immunotherapy, metastasis and recurrence of intermediate/advanced GC often lead to ultimate treatment failure [3, 4]. The prognoses of early GC and advanced gastric cancer are quite different [2, 5]. Therefore, it is urgent to find effective early biomarkers and related intervention targets to inhibit the progression of GC.

Circular RNAs (circRNAs) are a type of noncoding RNA produced by a noncanonical backsplicing event [6, 7]. circRNAs are characterized by evolutionary conservation and tissue- and cell-specific expression, and their biogenesis is regulated by specific cis-acting elements and trans-acting factors. Current studies on circRNAs have found that many circRNAs exert important biological effects by acting as microRNA sponges or binding to RNA binding proteins (RBPs), by regulating protein function or by being translated into proteins [8]. For instance, Yu et al. reported that hsa_circ_0003258 promotes prostate cancer metastasis by complexing with IGF2BP3 and sponging miR-653-5p [9]. Louis et al. and Chen al. reported a “one stone, two birds” phenomenon: circACTN4 promotes the progression of intrahepatic cholangiocarcinoma by recruiting YBX1 and activating the Hippo and Wnt/*β*-catenin pathway nexus [10, 11]. Gao et al. reported that circular RNA-encoded oncogenic E-cadherin variant promotes glioblastoma tumorigenicity through activation of the EGFR-STAT3 signal pathway [12]. In addition, an increasing number of studies have found that circRNAs are involved in the physiological and pathological processes of many diseases, including GC, and have various effects on biological processes such as proliferation, invasion, metastasis, and immunity [13]. Since circRNAs lack a 5′ cap and 3′ poly(A) tail, RNases have difficulty recognizing and degrading them [14]. Because of their highly stable structure, circRNA have strong potential as diagnostic, prognostic, and predictive biomarkers, highlighted by their detectability in liquid biopsy samples such as plasma, saliva, and urine. However, technical problems in detecting and evaluating circRNAs and gaps in biological knowledge need to be addressed, as well as the identification and characterization of more functional circRNAs and their possible regulatory mechanisms [13]. In order to advance this relatively young field of research and bring circRNAs to the forefront of clinical practice, it is therefore necessary to identify other circRNAs associated with GC to further our understanding of the underlying molecular mechanisms of GC.

Herein, we analyzed our microarray and identified a novel circRNA, circCASP9, that was downregulated in GC tissues and four GC cell lines. circCASP9 was determined to exert a tumor-suppressive effect on GC cell lines and nude mice. Based on predicted interactions among circRNAs, miRNAs, and mRNAs, we established a novel ceRNA network. Moreover, we proved that circCASP9 can sponge miR-589-5p to regulate the expression of KANK1. The identification of the circCASP9/miR-589-5p/KANK1 regulatory axis will help to elucidate the mechanisms underlying the occurrence and development of gastric cancer.

## 2. Materials and Methods

### 2.1. Clinical Specimens

We obtained 40 pairs of GC and paracancerous tissues from the First Affiliated Hospital of Chongqing Medical University between 2017 and 2020. None of the patients had received preoperative chemoradiotherapy. The tissue samples were immediately stored at −80°C after surgical resection and validated postoperatively by two pathologists. This study was approved by the Ethics Committee of Chongqing Medical University and was in compliance with the relevant guidelines (2021-26). Informed consent was obtained from all participants with GC.

### 2.2. Cell Culture

Human GC cell lines (AGS, MGC-803, and MKN-45), a human gastric epithelial cell line (GES-1), and a human embryonic kidney cell line (HEK293T) (Type Culture Collection of the Chinese Academy of Sciences, Beijing, China) were incubated in RPMI 1640 medium (VivaCell, Shanghai, China) or DMEM (Gibco, Carlsbad, USA) containing 10% fetal bovine serum (FBS) (VivaCell, Shanghai, China) at 37°C in a 5% CO_2_ atmosphere.

### 2.3. CircRNA Microarray Analysis

Our circRNA microarray containing five paired fresh GC and paracancerous tissues was manufactured by CapitalBio Technology (Beijing, China). In brief, total RNA was extracted with TRIzol reagent (Invitrogen, Carlsbad, CA, USA). circRNAs were enriched after the total RNA was digested with RNase R (Epicenter; Illumina Inc., San Diego, CA, USA). After amplification, the enriched circRNAs were transcribed into fluorescent cDNA. Then, this cDNA was hybridized using a hybridization oven (Agilent Technologies, G2545A). Next, the washed microarray was scanned by an Agilent Microarray Scanner (Agilent Technologies, G2565A). Finally, the microarray data were collected with Agilent Feature Extraction (version 10.7) and normalized with Agilent GeneSpring (Agilent Technologies, CA, USA).

### 2.4. RNA Extraction and qRT-PCR

Total RNA was isolated using TRIzol (TaKaRa, Japan) following the manufacturer's protocol. RNA was reverse transcribed into cDNA by using a PrimeScript RT Reagent Kit (RR037A; TaKaRa Bio Inc., Kusatsu, Japan) or a miRNA reverse transcription PCR kit (RiboBio). Quantitative real-time PCR was carried out using TB Green Premix Ex Taq II (RR820A; TaKaRa Bio Inc.). The divergent primers for amplification of circCASP9 were designed by GeneSeed (Guangzhou, China), and the other primers were purchased from Sangon Biotech (Shanghai, China). All primer sequences are shown in Table 1. Relative expression was determined using the 2^ΔΔ*Ct*^ method.

### 2.5. Plasmid Construction and Oligonucleotide Transfection

Cell lines with stable circCASP9 overexpression were established by transduction of the pHBLV-CMV crRNA lentiviral vector. The pHBLV-CMV crRNA lentiviral vector, into which the 286 bp circCASP9 cDNA sequence was inserted, was obtained from Hanbio Biotech (Shanghai, China). Stably transduced GC cells were screened with puromycin (2 mg/mL). The circCASP9-specific small interfering RNA (si-circCASP9) and normal control small-interfering RNA (si-NC) were designed by GenePharma (Shanghai, China). RiboFECT™ CP (RiboBio, Guangzhou, China) was used to transfect siRNAs as directed by the manufacturer. The silencing and overexpression efficiency of circCASP9 were verified with qRT-PCR. Lipofectamine 2000 (Invitrogen, Carlsbad, USA) was used for transfection according to the manufacturer's guidelines. The siRNA, miRNA mimic, and miRNA inhibitor sequences are listed in Table 2.

### 2.6. Fluorescence In Situ Hybridization (FISH) Assay

We used Cy3-labeled circCASP9 and Cy5-labeled miR-589-5p probes (GenePharma) in GC cells using FISH kits (RiboBio). Probes specific for circCASP9 and/or miR-589-5p were mixed and incubated overnight, and nuclei were then stained with 4′,6-diamidino-2-phenylindole (DAPI). Images were acquired using an LSM800 laser scanning confocal microscope (Carl Zeiss Microscopy GmbH, Jena, Germany). The sequences of the Cy3-labeled circCASP9 and Cy5-labeled miR-589-5p probes are listed in Table 2.

### 2.7. Cell Proliferation and Colony Formation Assays

Transfected GC cells (2 × 103 cells/well) were cultured in 96-well plates for 0, 24, 48, and 72 h and were then incubated with 10 *μ*L of CCK-8 reagent (MedChemExpress, Monmouth Junction, NJ, USA) for 2 h. The absorbance was measured at 450 nm. Transfected GC cells (6 × 102 cells/well) were cultured in 6-well plates for 8 d, fixed with paraformaldehyde (Servicebio, Wuhan, China), and stained with crystal violet (Solarbio Co. Ltd., Beijing, China). Then, colony formation was assayed using a CanoScan 9000F Mark II scanner (Canon Inc., Tokyo, Japan).

### 2.8. Ethynyl Deoxyuridine Incorporation Assay

Transfected GC cells (2 × 103 cells/well) were cultured in 96-well plates to the logarithmic growth phase. Cell viability was evaluated using Ethynyl Deoxyuridine (EdU) Labeling/Detection Kits (RiboBio), as described by the manufacturer. Briefly, 4% formalin was used to fix GC cells and 0.5% Triton X-100 was used to permeabilize GC cells. Then, Apollo solution and Hoechst 33342 solution were used for EdU incorporation and nuclear staining, respectively. Images were acquired using an LSM800 laser scanning confocal microscope (Carl Zeiss AG, Germany).

### 2.9. Migration and Invasion Assays

Transfected GC cells (3 × 104) were inoculated in a Transwell chamber (Corning Inc., Corning, NY, USA) for the migration assay or in a chamber precoated with 100 *μ*L of 1 mg/mL Matrigel matrix (BD Biosciences, San Jose, CA, USA) for the invasion assay. Then, cells in 300 *μ*L of serum-free medium were seeded in the upper chambers and 700 *μ*L of medium containing 10% FBS was added to the lower chambers. After 24 h of incubation, cells were fixed with paraformaldehyde, stained with crystal violet, and visualized using an inverted microscope (Leica).

### 2.10. Dual-Luciferase Reporter Assay

The wild-type (WT) or mutant-type (MUT) plasmids (pGL3-Firefly_Luciferase-Renilla_Luciferase) containing circCASP9 and the KANK1 3′-UTR were designed and constructed by Gene Create (Wuhan, China). HEK293T cells were cotransfected with the plasmid and miR-589-5p mimic, miR-4664-3p mimic, or miR-NC using Lipofectamine 2000. After incubation for 48 h, a Dual-Luciferase Assay Kit (Beyotime, China) was applied to perform the luciferase reporter assay.

### 2.11. Western Blotting Assay

Cells were collected and were then lysed using RIPA buffer (Beyotime, Shanghai, China). Samples were loaded onto an 8% SDS-PAGE gel and subsequently transferred onto a PVDF membrane (EMD Millipore, Billerica, MA, USA). After the membrane was blocked, we incubated it with primary antibodies specific for the reference gene GAPDH (1 : 6,000; ProteinTech, Wuhan, China) and KANK1 (1 : 2,000; ProteinTech, USA) at 4°C overnight. Next, the membrane was incubated with the corresponding secondary antibody (ProteinTech, Wuhan, China) for 2 h. Then, the membrane was visualized with enhanced chemiluminescence (ECL) solution (Advansta, CA).

### 2.12. Functional Enrichment Analysis of Target mRNAs

Overrepresented gene annotation terms in the biological process (BP), cellular component (CC), and molecular function (MF) categories were determined by gene ontology (GO) analysis [15]. Pathway enrichment was evaluated using the Kyoto Encyclopedia of Genes and Genomes (KEGG), with significance thresholds of *p* < 0.05 and *q* < 0.05 [16]. The results were visualized using the “clusterProfiler” R package [17].

### 2.13. Tumor Xenograft Assay

BALB/c nude mice (female, 4 weeks old, 5 in each group) were purchased from the National Laboratory Animal Center (Shanghai, China). MGC-803 cells were transduced with circCASP9 overexpression lentivirus or the NC vector, and the transduced cells were subcutaneously injected into the dorsum of BALB/c nude mice (2 × 106 cells/mouse) and the spleens of BALB/c nude mice (2 × 106 cells/mouse). Xenograft tumors were resected 5 weeks later for further analysis. The subcutaneous tumor size was measured weekly. The numbers of metastatic nodules were determined with a Leica inverted microscope (Leica, Wetzlar, Germany). All animal experiments were approved by the Animal Ethics Committee of Chongqing Medical University (2021-40).

### 2.14. Statistical Analysis

Data were analyzed GraphPad Prism (version 7, https://www.graphpad.com/) [18]. Group comparisons were assessed by Student's *t*-test (two-tailed) or analysis of variance (ANOVA). A heat map and volcano plot were generated using the “pheatmap” R package to visualize the differentially expressed circRNAs. Venn diagrams were constructed using the “Venn” R package to visualize the overlapping circRNAs and mRNAs. ^∗^*p* < 0.05 and ^∗∗^*p* < 0.01 were considered statistically significant.

## 3. Results

### 3.1. Identification and Characterization of circCASP9 in GC

**Figure 1:**
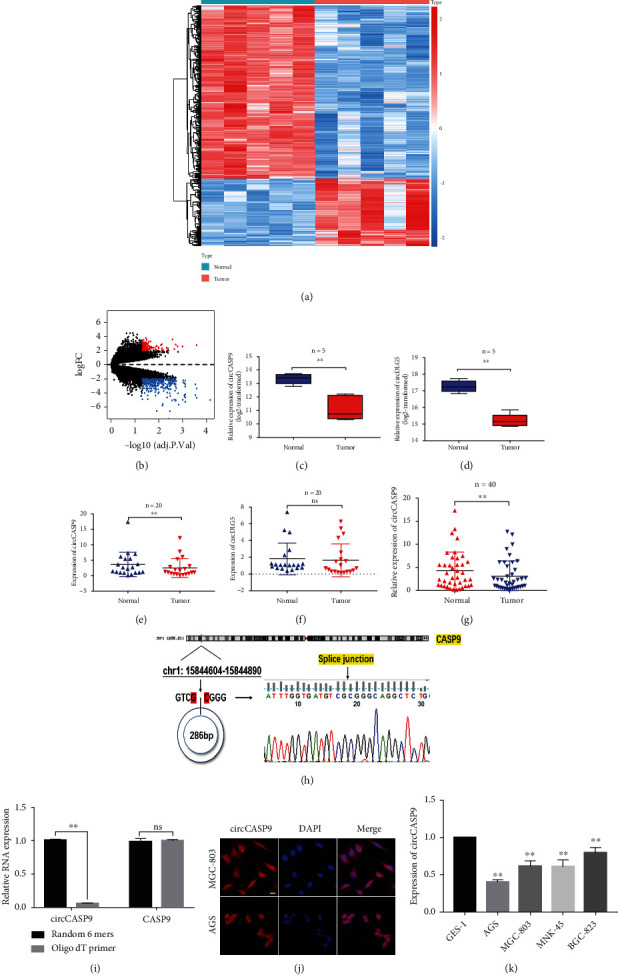
Identification of the differentially expressed circRNAs. (a) Heatmap for 158 downregulated circRNAs and 531 upregulated circRNAs in our circRNA microarray dataset. (b) Volcano map for 689 DECs in our circRNA microarray dataset. Red regions show upregulated circRNAs, blue regions show downregulated circRNAs, and the black regions show no statistically differential circRNAs (∣logFC | >2 and adj.*p* < 0.05). (c, d) Relative expression of (c) circCASP9 and (d) circDLG5 in circRNA microarray. (e, f) The expression of (e) circCASP9 and (f) circDLG5 in 20 paired GC tissues and adjacent normal tissues. (g) The expression of circCASP9 in 40 paired GC tissues and adjacent normal tissues. (h) circCASP9 locus location and the head-to-tail circular connection of circCASP9 were verified by Sanger sequencing. (i) The reverse transcription rate of Oligo dT primer and random primer to circCASP9. (j) FISH analysis of the localization of circCASP9 in AGS and MGC-803 cells. (k) The expression of circCASP9 in AGS, MGC-803, MNK45, and GES-1 cells. Values are shown as the mean ± standard error of the mean based on three independent experiments. ^∗^*p* < 0.05 and ^∗∗^*p* < 0.01.

Our circRNA microarray contained 170,420 circRNAs. A total of 689 DECs, namely, 531 downregulated and 158 upregulated circRNAs, were obtained from our microarray datasets (filtered by ∣logFC | >2 and adj.*p* (FDR) < 0.05) (Figures 1(a) and 1(b)). After excluding previously reported circRNAs, the two most significantly downregulated circRNAs, hsa_circ_0010039 (termed as circCASP9) and hsa_circ_0006649 (termed as circDLG5), were selected for further study (Figures 1(c) and 1(d)). To validate the differential expression of circCASP9 and circDLG5 between GC tissues and adjacent normal tissues, we collected 20 paired GC tissues and determined the expression of circCASP9 and circDLG5 by using qRT-PCR. The results indicated that circCASP9 but not circDLG5 was downregulated in tumor tissues compared with their normal counterparts (Figures 1(e) and 1(f)). Additional qRT-PCR results for 40 paired GC tissues also revealed downregulation of circCASP9 in tumor tissues (Figure 1(g)). Furthermore, Sanger sequencing was utilized to identify the circular structure of the circCASP9 molecule, which was formed by backsplicing (Figure 1(h)). The reverse transcription rate of circCASP9 with the oligo(dT) primer was significantly lower than that with the random primer, which also indicated the circular structure of circCASP9 (Figure 1(i)). Subsequently, FISH was performed to determine the localization of circCASP9. The results demonstrated that circCASP9 was located in both the cytoplasm and nucleus in MGC-803 and AGS cells (Figure 1(j)). We also determined the expression level of circCASP9 in multiple GC cell lines and found that it was significantly lower in AGS and MGC-803 cells than in GES-1 human gastric epithelial cells (Figure 1(k)). These results indicated that the circular RNA circCASP9 was downregulated in GC tissues and GC cell lines. Thus, circCASP9 was selected for further study.

### 3.2. Knockdown circCASP9 Promoted GC Cell Proliferation, Colony Formation, DNA Synthesis, Migration, and Invasion

**Figure 2:**
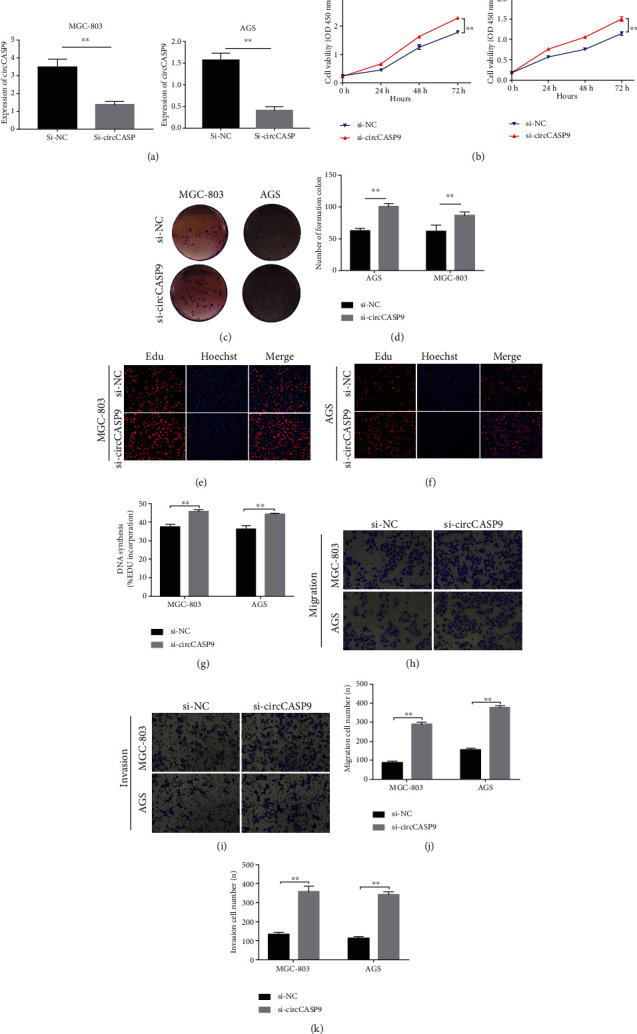
Knockdown circCASP9 promote proliferation, colony formation, DNA synthesis, migration, and invasion in MGC-803 and AGS cells. (a) The silence efficacy of si-circCASP9 in MGC-803 and AGS cells. (b) CCK-8 assay of MGC-803 and AGS cells transfected with si-NC and si-circCASP9. (c, d) Colony formation assay of MGC-803 and AGS cells transfected with si-NC or si-circCASP9. (e–g) EDU assay of MGC-803 and AGS cells transfected with si-NC or si-circCASP9. (h, j) Migration and (i, k) invasion ability of MGC-803 and AGS transfected with si-NC or si-circCASP9. Values are shown as the error of the mean based on three independent experiments. ^∗^*P* < 0.05 and ^∗∗^*P* < 0.01.

To confirm that dysregulation of circCASP9 could affect the biological function of GC cell lines, we first constructed a siRNA targeting circCASP9. qRT-PCR was utilized to determine the silencing efficiency of si-circCASP9 in MGC-803 and AGS cells (Figure 2(a)). Then, a CCK-8 assay was used to reveal that transfection of si-circCASP9 induced a significant increase in the proliferation capacity of AGS and MGC-803 cells compared to that of the negative control si-NC group (Figure 2(b)). In addition, colony formation assays demonstrated that AGS and MGC-803 cells transfected with si-circCASP9 had a higher colony-forming capacity (Figures 2(c) and 2(d)). EdU incorporation assays indicated that silencing circCASP9 caused a significant increase in the DNA synthesis capacity of AGS and MGC-803 cells (Figures 2(e)–2(g)). Transwell assays demonstrated that silencing circCASP9 promoted GC cell migration and invasion (Figures 2(h)–2(k)).

### 3.3. Overexpression of circCASP9 Suppresses GC Cell Proliferation, Colony Formation, DNA Synthesis, Migration, and Invasion

**Figure 3:**
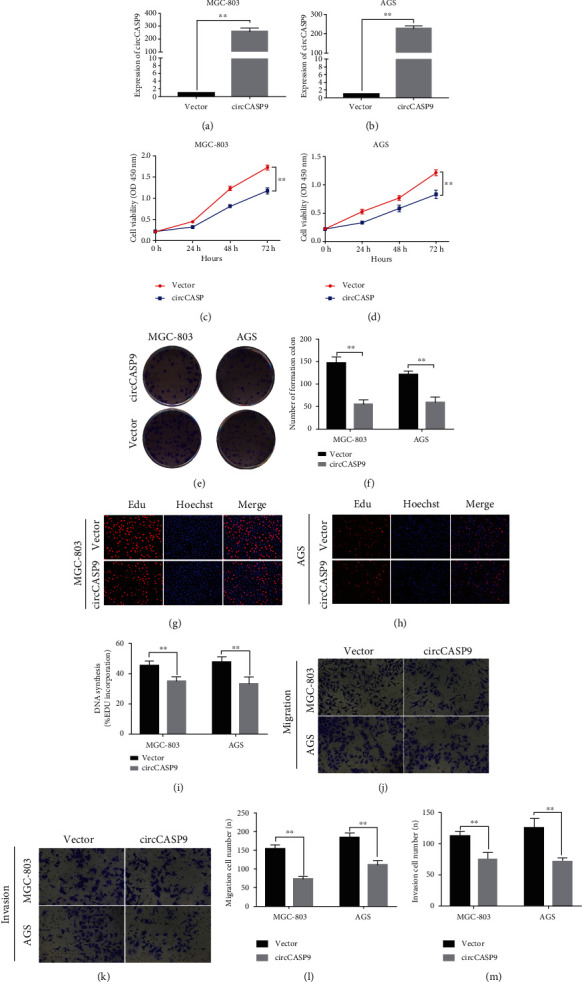
Overexpression of circCASP9 suppresses proliferation, colony formation, DNA synthesis, migration, and invasion in MGC-803 and AGS cells. (a, b) The overexpression efficacy of circCASP9 in (a) MGC-803 and (b) AGS cells. (c, d) CCK-8 and (e, f) colony formation detected the proliferation ability of MGC-803 and AGS cells transfected with vector and circCASP9. (g–i) EdU assay detected positive-stained cell percentage when transfected with vector and circCASP9. (j–m) Transwell analysis of the (j, l) migration and (k, m) invasion abilities of MGC-803 and AGS transfected with vector and circCASP9. Values are shown as the mean ± standard error of the mean based on three independent experiments. ^∗^*p* < 0.05 and ^∗∗^*p* < 0.01.

Considering that circCASP9 was downregulated in GC cell lines compared to the GES-1 cell line, we further generated MGC-803 and AGS cells with stable overexpression of circCASP9 and determined the effect of circCASP9 on biological functions in these GC cell lines. qRT-PCR was used to determine the overexpression efficiency of circCASP9 in AGS and MGC-803 cells (Figures 3(a) and 3(b)). Then, CCK-8 assays revealed that the proliferation ability of AGS and MGC-803 cells with stable overexpression of circCASP9 was lower than that of NC vector cells (Figures 3(c) and 3(d)). Colony formation assays demonstrated that circCASP9-overexpressing cells formed fewer and smaller colonies (Figures 3(e) and 3(f)). In addition, EdU incorporation assays indicated that the DNA synthesis ability of AGS and MGC-803 cells overexpressing circCASP9 was weaker than that of the NC groups (Figures 3(g)–3(i)). Similarly, the Transwell assay results suggested that circCASP9 overexpression inhibited GC cell migration and invasion (Figures 3(j)–3(m)). These results show that overexpression of circCASP9 can inhibit GC cell proliferation, migration, and invasion.

### 3.4. circCASP9 Acted as miR-589-5p Sponge

**Figure 4:**
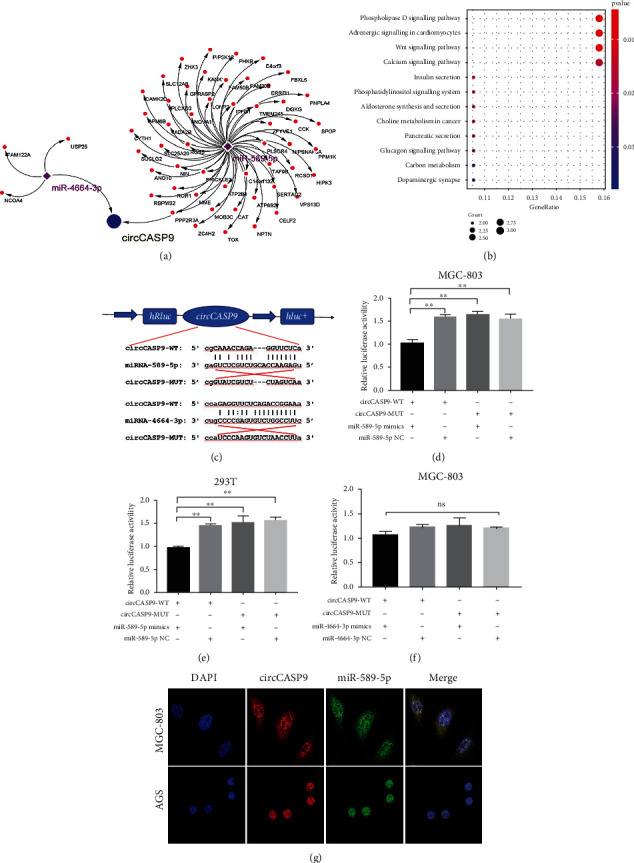
circCASP9 acted as a miR-589-5p sponge. (a) The circRNA-miRNA-mRNA regulatory network was established with one circRNA, two miRNA, and 55 mRNAs using Cytoscape. (b) The top 12 enrichment KEGG pathways involving the target genes. (c) Schematic representation of the potential binding sites of miR-589-5P and miR-4664-3p in the WT or MUT circCASP9. (d, e) Luciferase reporter assays verified the interaction between circCASP9 and miR-589-5P in (d) MGC-803 and (e) 293T cells. (f) Luciferase reporter assays show no interaction between circCASP9 and miR-4664-3P in MGC-803. (g) FISH analysis of the cellular colocalization of circCASP9 and miR-589-5P in MGC-803 and AGS. Nuclei were stained with DAPI (scale bar, 20 mm). Values are shown as the mean ± standard error of the mean based on three independent experiments. ^∗^*p* < 0.05 and ^∗∗^*p* < 0.01.

To further elucidate the mechanism underlying the antitumor function of circCASP9, we constructed a ceRNA regulatory network, which was composed of circCASP9, two miRNAs, and 55 mRNAs (Figure 4(a)). KEGG pathway enrichment analysis revealed that the 55 potential target genes regulated by circCASP9 were mainly involved in phospholipase D signaling, adrenergic signaling in cardiomyocytes, and Wnt signaling pathways (*p* < 0.01) (Figure 4(b)). In our ceRNA network, we identified two target miRNAs that might be sponged by circCASP9. To validate whether circCASP9 can interact with miR-589-5p and miR-4664-3p, we constructed luciferase plasmids expressing wild-type (WT) and mutant (MUT) circCASP9 (Figure 4(c)). Luciferase reporter assays proved that the miR-885-5p mimic significantly decreased the luciferase activity of circCASP9-WT but not of circCASP9-MUT in MGC-803 (Figure 4(d)) and 293T (Figure 4(e)) cells compared with the corresponding miR-NC groups. However, we observed that the miR-4664-3p mimic did not affect the luciferase activity of circCASP9-WT (Figure 4(f)). To further explore the relationship between circCASP9 and miR-589-5p, we determined the localization of circCASP9 and miR-589-5p by using FISH. The results demonstrated that miR-589-5p and circCASP9 were colocalized in the cytoplasm of MGC-803 and AGS cells (Figure 4(g)). These results indicated that circCASP9 might function as a sponge of miR-589-5p to affect GC progression.

### 3.5. miR-589-5p Reversed the Inhibitory Effects of circCASP9 on Proliferation and Invasion

**Figure 5:**
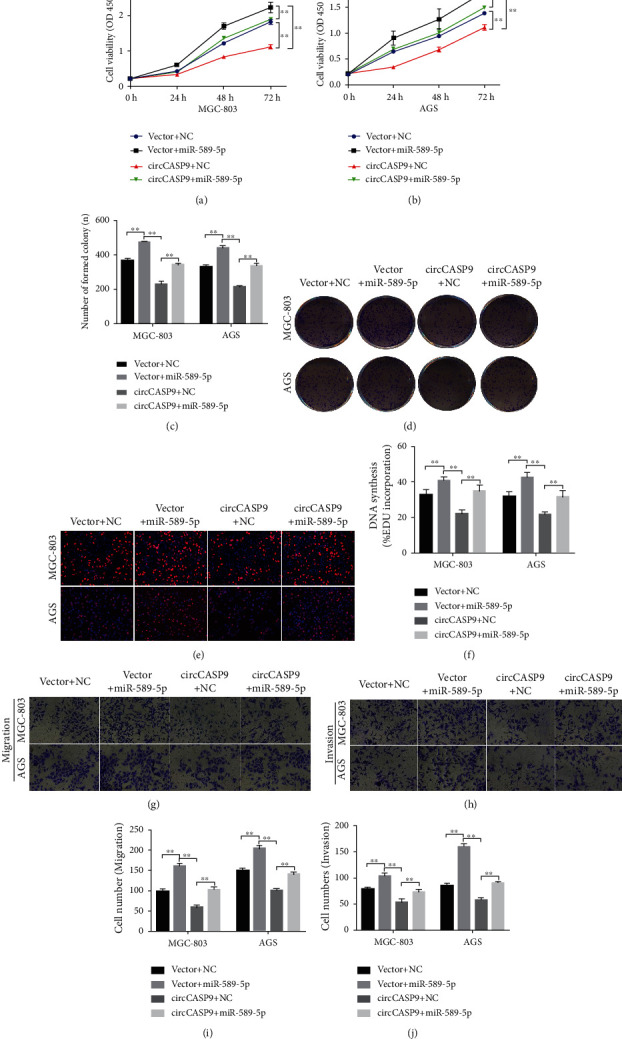
miR-589-5P reversed circCASP9-induced decrease in dell proliferation, migration, and invasion in GC cells. (a, b) CCK-8 analysis of cell viability after the cotransfection of miR-589-5P mimics and circCASP9 in MGC-803 and AGS cells. (c, d) Colony formation analysis of colony numbers after the cotransfection of miR-589-5P mimics and circCASP9 in MGC-803 and AGS cells. (e, f) EDU assay detected positive-stained cell percentage after the cotransfection of miR-589-5P mimics and circCASP9 in MGC-803 and AGS cells. (g–j) Transwell assays of the cell (g, i) migration and (h, j) invasion potential after the cotransfection of miR-589-5P mimics and circCASP9 in MGC-803 and AGS cells. Values are shown as the mean ± standard error of the mean based on three independent experiments. ^∗^*p* < 0.05, ^∗∗^*p* < 0.01.

To explore whether miR-589-5p is responsible for the functional alterations mediated by circCASP9, we cotransfected circCASP9 and miR-589-5p into MGC-803 and AGS cells. CCK-8 and colony formation assays were conducted to determine the proliferation ability of AGS and MGC-803 cells. The results demonstrated that miR-589-5p significantly increased the proliferation ability of GC cells. However, circCASP9 attenuated the increase in proliferation ability induced by miR-589-5p (Figures 5(a)–5(d)). Similarly, circCASP9 decreased the increase in DNA synthesis ability mediated by miR-589-5p (Figures 5(e) and 5(f)). Regarding migration and invasion, we found that exogenous upregulation of miR-589 expression enhanced the migration and invasion abilities of GC cells. Additionally, circCASP9 partially reversed the increases in the migration and invasion abilities of MGC-803 and AGS cells caused by miR-589-5p mimic transfection (Figure 5(g)–5(j)).

### 3.6. The circCASP9/miR-589-5p Axis Directly Affects KANK1 in GC

**Figure 6:**
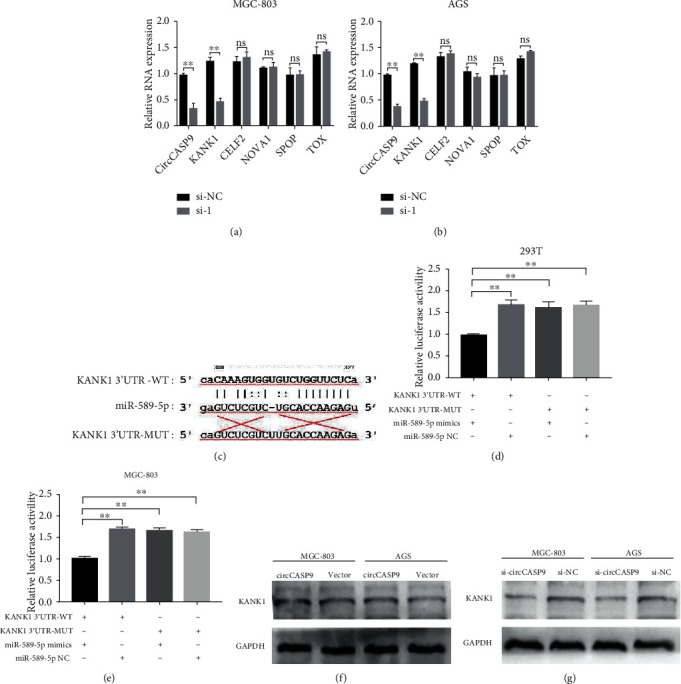
KANK1 may be the target gene of miR-589-5p. (a, b) KANK1 had low expression after knockdown of circCASP9 in (a) MGC-803 and (b) AGS cells. (c) Schematic representation of the potential binding sites of miR-589-5p in the WT or MUT KANK1 3′UTR. (d, e) The interaction between miR-589-5P and KANK1 was verified by dual-luciferase reporter assay in (d) 293T and (e) MGC-803 cells. (f, g) Western blot analysis of the expression of KANK1 after (f) overexpression or (g) knockdown circCASP9 in MGC-803 and AGS cells. Values are shown as the mean ± standard error of the mean based on three independent experiments. ^∗^*p* < 0.05 and ^∗∗^*p* < 0.01.

CircCASP9 could affect the proliferation, migration, and invasion of GC cells by sponging miR-589-5p. To understand the potential mechanism mediated by the circCASP9/miR-589-5p axis, we analyzed the expression of five genes (KANK1, CELF2, NOVA1, SPOP, and TOX) closely associated with GC progression. qRT-PCR results suggested that silencing circCASP9 could cause a decrease in KANK1 mRNA expression in MGC-803 and AGS cells (Figures 6(a) and 6(b)), indicating that the circCASP9/miR-589-5p axis might exert an antitumor effect by affecting KANK1 expression. To verify our hypothesis, we constructed KANK1 3′-UTR-WT and 3′-UTR-MUT (without miR-589-5p binding sites) luciferase vectors and conducted luciferase reporter assays (Figure 6(c)). The results of these luciferase reporter assays showed that the luciferase activity of KANK1 3′-UTR-WT was markedly reduced by the miR-589-5p mimic in both HEK293T and MGC-803 cells, while the luciferase activity of KANK1 3′-UTR-MUT was not altered by the miR-589-5p mimic compared with that in the corresponding control groups (Figures 6(d) and 6(e)). We also determined whether circCASP9 can affect the protein level of KANK1 by using Western blot analysis. As expected, overexpression of circCASP9 led to an increased protein level of KANK1; however, knockdown of circCASP9 decreased KANK1 protein expression in both MGC-803 and AGS cells (Figures 6(f) and 6(g)). These results suggest that the circCASP9/miR-589-5p axis might exert a tumor-suppressive effect by mediating KANK1 expression.

### 3.7. circCASP9 Inhibited GC Tumor Growth and Liver Metastasis In Vivo

**Figure 7:**
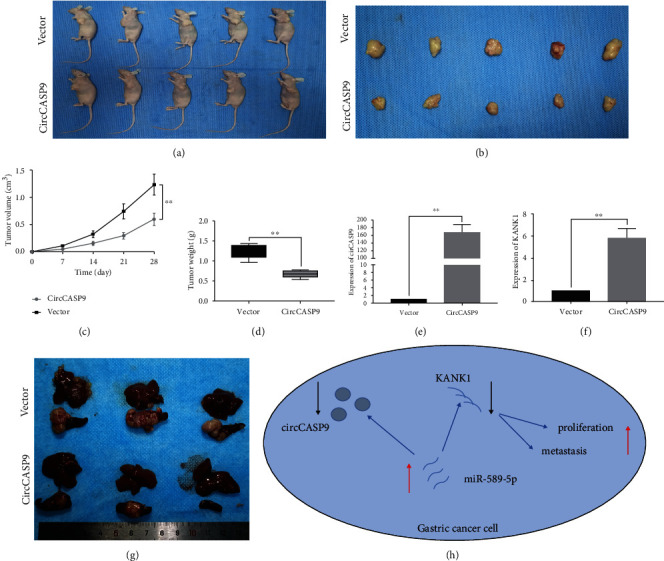
The effects of circCASP9 on tumor growth in vivo. (a, b) The tumor size of xenograft tumors induced by circCASP9 overexpression or NC vector in transfected MGC-803 cells. (c) A growth curve analysis of tumor growth in MGC-803 cells transfected with the circCASP9 overexpression vector or NC vector. (d) The tumor weight of xenograft tumors induced by circCASP9 overexpression or NC vector in transfected MGC-803 cells. (e, f) The expression of circCASP9 and KANK1 induced by circCASP9 overexpression or NC vector in transfected MGC-803 cells. (g) The metastasis nodes of xenograft tumors induced by circCASP9 overexpression or NC vector in transfected MGC-803 cells. (h) Schematic representation of the circCASP9 mechanism in GC cells. Values are shown as the mean ± standard error of the mean based on three independent experiments. ^∗^*p* < 0.05 and ^∗∗^*p* < 0.01.

To further explore whether circCASP9 affects tumor proliferation in vivo, we injected MGC-803 cells with stable overexpression of circCASP9 or the NC vector into subcutaneous tissues of nude mice to establish a xenograft tumor model. The results of xenografting revealed that circCASP9 overexpression significantly inhibited the growth of tumors formed by GC cells in vivo (Figures 7(a) and 7(b)). The volume and weight of tumors were markedly reduced in the circCASP9 overexpression group compared with the NC vector group (Figures 7(c) and 7(d)). Consistent with the in vitro observations, circCASP9 overexpression upregulated the expression of KANK1 (Figures 7(e) and 7(f)). We also established a tumor metastasis model to determine whether circCASP9 can inhibit the metastasis of GC cells in vivo. As expected, the nude mice injected with circCASP9-overexpressing cells have smaller and fewer liver metastatic nodules than the mice in the vector group (Figure 7(g)). Overall, these findings demonstrated that circCASP9 acts as a sponge of miR-589-5p and upregulates KANK1, thereby inhibiting the progression and metastasis of GC (Figure 7(h)).

## 4. Discussion

Circular RNAs were originally regarded as noncoding RNAs produced by splicing errors. However, recently, circRNAs have been identified as a large class of endogenous, conserved noncoding RNAs produced by backsplicing [6, 7]. circRNAs are a kind of noncoding RNA with a covalently closed circular structure, which means that circRNAs are more stable than linear RNAs [19]. Thus, many circRNAs have been used as diagnostic biomarkers or prognostic factors in multiple tumors, including gastric cancer [20, 21]. circRNAs have also been reported to participate in a wide range of biological and pathological processes, an observation that led to a major focus of recent research on circRNAs [22].

Circular RNAs have been proven to exert crucial regulatory effects on various biological activities, particularly tumorigenesis and tumor progression [23–25]. Cytoplasmic circRNAs have been reported to act as sponges of miRNAs, thereby modulating the expression of target genes associated with tumor progression [26]. For example, circCCDC9 can suppress the development of GC via the miR-6792-3p/CAV1 axis [27]. circLMTK2 can lead to GC growth and metastasis by regulating the miR-150-5p/c-Myc axis [28]. circLARP4 inhibits GC development by sponging miR-424-5p [29]. Although a large number of circRNAs have been identified to be involved in GC progression [30–32], the mechanisms by which circRNAs contribute to GC remain largely unknown.

In this study, we identified a novel circular RNA, circCASP9, and proved that circCASP9 is downregulated in GC tissue samples and cell lines, consistent with the data from our circRNA microarray analysis. circCASP9 is produced from exon 10 of CASP9 (caspase 9). circCASP9's parental gene, CASP9, has mainly been reported to participate in intrinsic apoptosis as well as macroautophagy/autophagy regulation [33]. To our knowledge, this is the first study to identify a close relationship between circCASP9 and GC. We performed a series of functional assays in vitro and in vivo to investigate the role of circCASP9 in GC. We found that overexpression of circCASP9 significantly reduced the proliferation, migration, and invasion abilities of GC cells in vitro, as well as tumor growth and metastasis in vivo, whereas silencing of circCASP9 had the opposite effects. These findings demonstrated that circCASP9 might be a novel promising biomarker in GC cells and might be associated with GC tumorigenesis and development.

Many cytoplasmic circRNAs exert tumor-promoting or tumor-suppressing effects by sponging target miRNAs [34, 35]. To elucidate the potential mechanism by which circCASP9 affects GC progression, we constructed a ceRNA network composed of circCASP9, two miRNAs, and 55 mRNAs. Subsequently, we constructed WT and MUT luciferase plasmids and determined the effect of miR-589-5p on luciferase activity. The results of the dual luciferase assays showed that circCASP9 can sponge miR-589-5p and prevent the binding of miR-589-5p to its target genes. In addition, our FISH results indicated that circCASP9 can interact with miR-589-5p. miR-589-5p has been found to exert crucial effects on multiple tumors. For instance, miR-589-5p can reduce stemness characteristics in CD90+ CSCs in hepatocellular carcinoma [36]. LOXL1-AS1 can promote laryngeal carcinoma progression by sponging miR-589-5p [37]. The lncRNA LOXL1-AS1 exerts oncogenic effects on renal cell carcinoma by sequestering miR-589-5p [38]. However, the function of miR-589-5p has not been previously reported in GC. To explore whether miR-589-5p functions in GC development and whether miR-589-5p is associated with the antitumor function of circCASP9, we performed rescue experiments. The results demonstrated that miR-589-5p exerts a tumor-promoting effect, while cotransfection of circCASP9 partially reversed the effect of miR-589-5p. These results suggest that circCASP9 might exert a tumor-suppressive effect by sponging miR-589-5p.

In our ceRNA network, we identified a total of 52 downstream target genes of miR-589-5p. KEGG pathway enrichment analysis revealed that these 52 genes were mainly enriched in twelve signaling pathways, including those associated with tumor-related processes. The phospholipase D signaling pathway can affect tumorigenesis [39]. The activated WNT signaling pathway has been reported to promote GC cell proliferation, metastasis, and invasion [40, 41]. The activated calcium signaling pathway can inhibit non-small cell lung cancer cell proliferation and promote apoptosis [42]. Choline metabolism has also reported to be associated with tumorigenesis and might be a therapeutic target [43]. To identify the target genes of the circCASP9/miR-589-5p axis, we performed qRT-PCR and found that silencing circCASP9 significantly inhibited the mRNA expression of the KN motif and ankyrin repeat domains 1 (KANK1). KANK1 has been reported to participate in tumorigenesis and tumor progression in various cancers. circDDX17 can affect sensitivity to 5-fluorouracil by regulating the miR-31-5p/KANK1 axis [44]. In addition, TRAIP-mediated downregulation of KANK1 can enhance the invasion and proliferation of osteosarcoma cells through the IGFBP3/AKT pathway [45]. KANK1 has also been reported to exert a tumor-suppressive effect by regulating the Wnt/*β*-catenin/Axin2 pathway in GC development [46]. To validate KANK1 as a target of miR-589-5p, we constructed plasmids expressing the WT and MUT 3′-UTRs of KANK1. The dual-luciferase assay indicated that KANK1 might be the direct target gene of miR-589-5p. In addition, Western blot analysis showed that circCASP9 can promote the protein expression of KANK1 in GC cells. To extend our findings in vitro, we validated the function of circCASP9 *in vivo*. As expected, overexpression of KANK1 significantly inhibited GC tumor growth and metastasis. Collectively, these results indicate that the circCASP9/miR-589-5p axis can modulate GC progression by affecting KANK1 expression.

Despite the positive findings in our study, we recognize that our study has several limitations. The association between circCASP9 expression and prognosis could not be clinically validated because of an insufficiency of clinical samples with adequate follow-up. In addition, although KANK1 has been reported to participate in GC progression by modulating the WNT pathway, we did not validate whether the circCASP9/miR-589-5p/KANK1 axis can affect GC progression through the WNT pathway.

In conclusion, our findings indicated that circCASP9 plays a crucial role in GC development. circCASP9 can act as a sponge of miR-589-5p to regulate the expression of KANK1, thereby affecting GC progression. The circCASP9/miR-589-5p/KANK1 axis might contain a useful target for further investigation into the occurrence and development of GC.

## Figures and Tables

**Table 1:** Quantitative real-time PCR primer sequences.

Name	Primer	
circCASP9	F: AGGATTTGGTGATGTCGCGG	R: TGTCCTCTAAGCAGGAGATG
circDLG5	F: CAGAAGGAGATCGGTGACCT	R: CGCACGCACTGGATCTTC
KANK1	F: TCGAGGAAAAAGGTTGACAAAGC	R: TCCACCAGGTCCATGTGACT
CELF2	F: GCGTTCAGCGGCATTCAAC	R: AGAGAGAGGGTTTGCATTGGT
NOVA1	F: TACTGAGCGAGTGTGCTTGAT	R: GTCTGGGGTTGTAGAATGCTG
SPOP	F: GCCCCGTAGCTGAGAGTTG	R: ACTCGCAAACACCATTTCAGT
TOX	F: TATGAGCATGACAGAGCCGAG	R: GGAAGGAGGAGTAATTGGTGGA
CASP9	F: CTCAGACCAGAGATTCGCAAAC	R: GCATTTCCCCTCAAACTCTCAA
GAPDH	F: CTTTGGTATCGTGGAAGGACTC	R: GTAGAGGCAGGGATGATGTTCT

**Table 2:** Nucleotide sequences of siRNAs and probes.

Name	Sequences
Si-circCASP9-1	ATTTGGTGATGTCGCGGGC
Si-circCASP9-2	GATTTGGTGATGTCGCGGG
Si-circCASP9-3	TTTGGTGATGTCGCGGGCA
miR-589-5p mimic	UGAGAACCACGUCUGCUCUGAG
miR-4464-3p mimic	CUUCCGGUCUGUGAGCCCCGUC
Mimic NC	UUUGUACUACACAAAAGUACUG
Inhibitor NC	CAGUACUUUUGUGUAGUACAAA
FISH probe of circCASP9	CC+TGCCCGCGACA+TCACCAAATC
FISH probe of miR-589-5P	CTCAGAGCAGACG+TGGT+TCTCA

## Data Availability

The data used to support the findings of this study are included within the article.
